# Element contents and their seasonal dynamics in leaves of alder *Alnus glutinosa* (L.) Gaertn

**DOI:** 10.1007/s10661-024-12367-x

**Published:** 2024-02-01

**Authors:** Petr Hrdlička, Emanuel Kula

**Affiliations:** 1 Department of Chemistry and Biochemistry, Faculty of AgriSciences, Mendel University in Brno, Zemědělská 1665/1, 613 00 Brno, Czech Republic; 2 Department of Forest Protection and Game Management, Faculty of Forestry and Wood Technology, Mendel University in Brno, Zemědělská 810/3, 613 00 Brno, Czech Republic

**Keywords:** Deciduous tree, Macroelements, Microelements, Non-nutrient elements, Ore Mountains, Growing season

## Abstract

*Alnus glutinosa* is an actinorhizal plant that fixes N via actinomycetes. Compared to other trees, *A. glutinosa* is more resistant to environmental stress and able to uptake soil nutrients more easily. *Alnus glutinosa* grows well not only in natural stands but also in degraded environment or soil in need of restoration. Changes in the contents of selected macro-, micro-, and non-nutrient elements in the leaves of *A. glutinosa* during the vegetation season were monitored in the Ore Mountains (Czech Republic), an area affected by extreme air pollution in the past. Decreased foliar content of N, P, K, and Cu, and increased content of Ca, Mn, Zn, and Al were observed; the content of other elements (S, Mg, Pb, and Cd) varied during the growing season or remained constant. From the viewpoint of nutrition, the content of N, S, Ca, and Mg macroelements was adequate; concentrations of P and K were low. Excessive amounts of Mn and Zn were measured, and the level of Cu was good. Non-nutrient elements Pb and Cd were present at the background level, and the level of Al was high. N/P, N/Ca, N/Mg, and Ca/Mg ratios were balanced, S/N value showed the lack of S, and N/K ratio indicated low content of K, which caused also suboptimal K/Ca and K/Mg values. The P/Al ratio varied from balanced to lower values. The content of individual elements and monitored changes were influenced by the amount of elements in the soil, moisture conditions, foliage phenology, and altitude.

**Supplementary Information:**

The online version contains supplementary material available at 10.1007/s10661-024-12367-x.

## Introduction

*Alnus glutinosa* (L.) Gaertn. (black alder) is a woody plant known for its ability to improve soil quality. This actinorhizal plant uses symbiotic actinomycetota Frankia, forming nitrogen-fixing root nodules (Bélanger et al., 2013), as well as vesicular-arbuscular mycorrhizae and ectomycorrhizae for nutrition purposes (Harley & Harley, 1987; Jha et al., 1993). In disturbed forest ecosystems, *A. glutinosa* is an important pioneer species (Wolfe et al., 2022). Compared to other trees, it is more resistant to environmental stress and able to uptake soil nutrients, especially nitrogen and phosphorus (P), more easily (Monzón & Azcón, 2001), but requires sufficient soil moisture. Besides its natural habitat, *A. glutinosa* can grow also in disturbed environments (Oliveira et al., 2005), helping to restore the quality of disturbed soil in degraded localities (Świątek et al., 2019). Being highly frost-tolerant at the same time (DeWald & Steiner, 1986), *A. glutinosa* is suitable as a substitute woody plant for wet sites in previously air-polluted mountain areas (e.g., Ore Mountains, Czech Republic), where it can grow as a healthy specie, resistant to insect pests and fungal pathogens (Slodičák et al., 2008). The use of *A. glutinosa* as a suitable restoration tree has been reported (Kacálek et al., 2017; Orfanoudakis et al., 2010).

Generally, the growth of forest trees depends on site conditions (nutrient content in the soil, their form, availability, and development of mycorrhiza or actinorhiza). Assimilation occurs to a smaller extent, i.e., SO_2_ as a source of sulfur (S) (De Kok, 1990; Gebauer et al., 1994; Slovik, Heber, et al., 1992; Slovik, Kaiser, & C., K., Kindermann, G.,, & Heber, U., 1992; Tausz et al., 1998). The content of nutrients in individual organs of the tree varies in a wide range during the growing season. The amount of elements in the leaves of individual tree species changes differently during a vegetation period according to the soil type and quality, moisture level, character, and intensity of the pollution burden. The phenology of the leaves and their development at the beginning of the growing season also play an important role (Hájková et al., 2012).

The content of elements in assimilatory organs is varied during the growing season according to the current physiological needs of a tree and the mobility of elements. Mobile elements, like N, P, magnesium (Mg), and potassium (K), are redistributed into other parts of the tree, while partially mobile S, zinc (Zn), copper (Cu), and immobile ones like calcium (Ca) and manganese (Mn) can be accumulated in assimilation apparatus. Redistribution of some nutrients from mature to newly developing leaves in the course of the vegetation period or their deposition in various organs at the end of the vegetation season can be significant (Marschner, 2006).

The content and distribution of chemical elements in leaves, as well as the balanced ratio of elements (w/w), are important parameters for the evaluation of nutritional status, influence of emissions, and overall health of woody plants; evaluation methodology has been described in detail in previous studies (Hrdlička & Kula, 2004, 2009, 2011).

The aim of this study was to ascertain (*i*) the total content of selected elements in the soil; (*ii*) the changes in the contents of macroelements, S, N, P, Ca, Mg, and K, microelements, Mn, Zn, and Cu, and non-nutrient elements, lead (Pb), cadmium (Cd), and (Al) in the leaves of *A. glutinosa* during the growing season; (*iii*) tree nutrition status (at the end of growing season); (*iv*) changes in the ratio of selected elements (mainly ratios to N) during the growing season and at the end of growing season; and (*v*) similarity of monitored localities by cluster analysis.

## Material and methods

### Research area

**Fig. 1:**
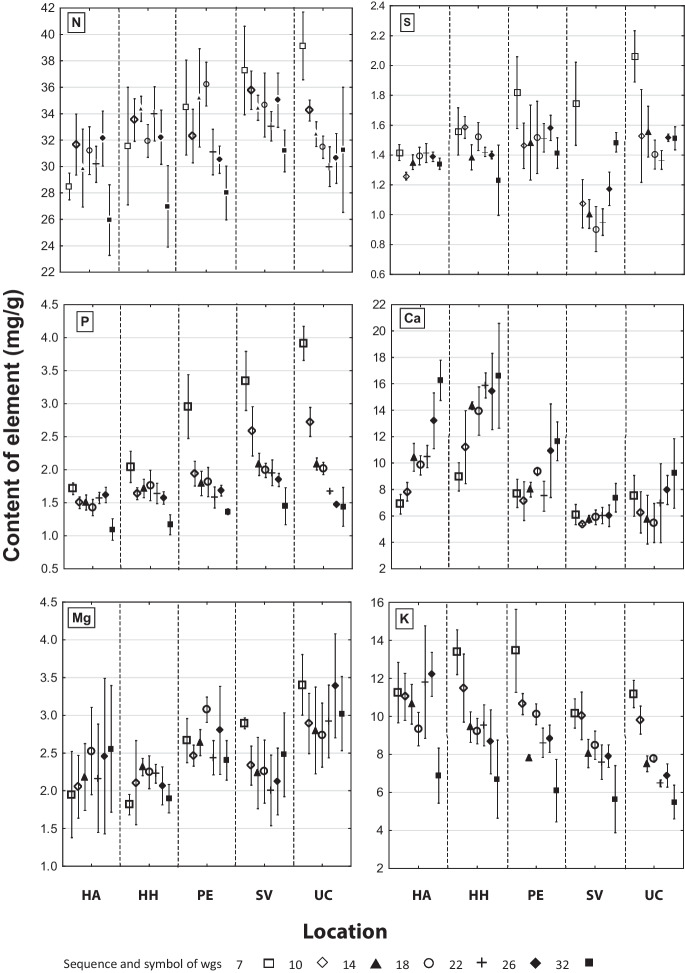
The content of macroelements in *Alnus glutinosa* leaves (mean ± confidence interval; according to the week of growing season - “wgs”)

Five localities of different altitudes, forest site complexity, and air pollution level in the northwestern part of the Czech Republic, in eastern Krusne hory (“Ore Mountains”) and the Decin sandstone highlands, were selected as the research area (Table 1). A long-term trend of reduced SO_2_ and NO_x_ concentrations was monitored in the area under investigation during the years 2005–2009 (Czech Hydrometeorological Institute, 2011) (Suplementary material Table S1); the data for SO_2_ and NO_x_ from 2009 are given as a chart. The lowest monthly concentrations of SO_2_ and NO_x_ were measured during the growing season from March to September, with SO_2_ levels < 5–10 μg m^−3^ (June to August, fast-growing season) and NO_x_ levels < 10–15 μg m^−3^ (Suplementary material  Fig. 1a and 1b). A negative effect of these emissions on the stands is not expected.

**Table 1:** Localities—general characterization

Loc (mark)	WRB (^1^)	FSC	Age	T [°C]	P [mm]	Alt [m a.s.l.]	NL	EL
Haj (HA)	Fluvisols (FLq)	2H	47	7.5–8.0	540	295	50° 37′ 16″	13° 43′ 14″
Horni Haj (HH)	Cambisols (KAd)	4N	19	6.5–7.5	815	460	50° 38′ 23″	13° 41′ 42″
Petrovice^2^ (PE)	Histosols (ORFi)	6G	11	5.0–6.0	780	670	50° 46′ 17″	13° 57′ 36″
Svahova (SV)	Gleysols (GLo)	7G	30	4.0–4.5	890	790	50° 33′ 55″	13° 24′ 26″
U chat (UC)	Podzols (PZm)	8G	25	4.0–4.5	1100	845	50° 41′ 26″	13° 41′ 09″

Notes: *Loc* localities, *WRB* World Reference Base—see Zádorová and Penížek (2011), *FSC* forest site complex—see Viewegh et al. (2003), *T* annual mean temperature, *P* annual mean precipitation (2005–2009), *Alt* altitude, *NL* north latitude, *EL* east longitude

(^1^) In parentheses: Czech Taxonomic Classification System of Soils and Czech Soil Map (Czech Geological Survey. Soil map, 2021; Czech Office for Surveying. Czech National Geoportal, 2020; Němeček et al., 2011; Zádorová & Penížek, 2011)

^2^During the sampling, this site was heavily waterlogged and flooded with water

### Soil characteristics in individual locations

Basic characteristics of the soils according to the World Reference Base (WBR) (Czech Geological Survey. Soil map*,* 2021*;* Czech Office for Surveying. Czech National Geoportal*,* 2020) classification in individual sites are given in Table 1. Habitat conditions at examined localities are defined by the forest site complex (FSC) (Viewegh et al., 2003). The site Haj (2H - *Fageto-Quercetum illimerosum mesotrophicum*) is a lowland habitat (200–400 m above sea level); the soil is deep, loamy, loose, showing signs of drought stress and buffeting, but not degradation. Horni Haj (4 N - *Fagetum lapidosum acidophilum*) is a hilly habitat (350–500 m a.s.l.); compared to the first site, the soils are more poor and acidic, moderately deep, moist, and very skeletal. Petrovice (6G - *Piceeto-Abietum paludosum mesotrophicum*) is a highland (550–700 m a.s.l.), permanently waterlogged habitat with humic soils, and *A. glutinosa* in a natural stand. The site Svahova (7G - *Abieto-Piceetum paludosum mesotrophicum*) is a highland (650–950 m a.s.l.), waterlogged habitat with loamy-sandy soils and *A. glutinosa* in a natural composition of species. The site U Chat (8G - *Piceetum paludosum mesotrophicum*) is a typical Ore Mountain plateau habitat (900–1050 m a.s.l.) with deep soils on acidic subsoil, skeletal and moist. The soil characteristics were specified based on a determination of some elements.

### Soil sampling and analysis

Three soil pits were excavated at each site to describe the soil profile and delineate individual horizons, the topsoil horizon with accumulated organic matter (Ah), and in the subsoil (B). Soil analysis was carried out by direct measurement in the field using an XRF analyzer after exposing the relevant horizon. Subsequently, soil samples (Ah and B) were collected, air-dried, and homogenized before laboratory analysis. The total contents of selected elements were established by a handheld X-ray fluorescence analyzer (Olympus Innov-X Delta), a device for a very quick in situ analysis of various elements in soils. The measurement was performed at 4 W with 200 μA (max) X-ray tube as a source of radiation (Delta X-ray, 2013). XRF spectrometry was used to determine the total content of S, P, Ca, K, Mn, Zn, Cu, Pb, and Al. The method is not suitable for the determination of N content; Mg and Cd contents were below the detection limit.

### Leave sampling and analysis

*Alnus glutinosa* stands were monitored in the selected localities of the eastern Ore Mts. (295–845 m a.s.l.) (Table 1). Leave samples were taken in monthly intervals throughout the growing season of 2009 (May to October), divided into early-growing season (May–June), fast-growing season (July–September), and late-growing season (October) (comp. Li et al. (2017)). Using telescopic shears with a reach of up to 12 m, one branch from the lower and the other from the middle of the sun-exposed parts of each tree crown was cut from 10- to 50-year-old trees of *A. glutinosa* (usually in 3 m and 7 m heights, except the locality HH, where the samples were taken from 5 m and 12 m). Fully developed leaves (visual control) from both branches were stored as a mixed sample in paper bags. One hundred five samples were taken from 15 trees in five stands of *A. glutinosa*. Before the analysis, the samples were dried for 24 h at 70 °C and turned to the ashes in a muffle furnace at 200 °C (2 h) and at 450 °C (12 h).

Mineral residues were extracted with HCl (1 mol l^–1^). K was determined by flame atomic emission spectrophotometry, Ca, Mg, Mn, Zn, Cu, Pb, and Al by absorption spectrophotometry, and Cd by ETAAS using an AAS 30 (Carl Zeiss, Jena, Germany) spectrometer. S and N were determined by classical methods using the LECO analyzer and Tecator Digestion system 20. P was determined spectrophotometrically using APHA Standard Method 4500-P C (1992) after wet digestion (H_2_SO_4_, H_2_O_2_, and HgO) according to Hrdlička and Kula (1998). All determinations were carried out by an accredited laboratory (Laboratory-Morava, 2010).

### Data evaluation

Because of the fact that both *A. glutinosa* and *Betula pendula* Roth belong to the family Betulaceae, similar requirements for nutrient uptake and leaf element content levels were expected. While *A. glutinosa* differs from other trees in the mechanism of N uptake in the form of actinorhiza, the uptake of other elements is similar (Millett et al., 2012). At the end of the growing season, however, birch transports N into the wood, which *A. glutinosa* does not because of the actinorhizae (Rodriguez-Barrueco et al., 1984).

Nutritional level of *A. glutinosa* is based on the content of selected elements. In the soil profile, horizons Ah and B were selected for element monitoring (Suplementary material Table S2, mg kg^−1^). In the leaves, macroelements (N, S, P, Ca, Mg, and K; mg g^−1^), microelements (Mn, Zn, Cu; μg g^−1^), and non-nutrient elements (Pb, Cd, Al; μg g^−1^) were determined. The nutrient classification was performed according to *A. glutinosa* leaf data obtained at the end of the growing season (UN-ECE, 2016). Limit values (Tables 2 and 3) were determined according to the classification for birch, using the designation for the ratios based on Stefan et al. (1997).

**Table 2:** Classification values for *Alnus glutinosa*

N [mg g^−1^]	S [mg g^−1^]	P [mg g^−1^]	Ca [mg g^−1^]	Mg [mg g^−1^]	K [mg g^−1^]
25–40	1.3–2	1.5–3	3–15	1.5–3	10–15
Mn [μg g^−1^]	Zn [μg g^−1^]	Cu [μg g^−1^]	Pb [μg g^−1^]	Cd [μg g^−1^]	Al [μg g^−1^]
30–100	15–50	6–12	2–10	0.1–0.2	103–120

Adequate or optimum range of concentrations for macro- and microelements: lower values are too low/deficient; higher values are too high or surplus. For non-nutrient (toxic) elements (Pb, Cd, and Al), given values are medium: lower values are low (background level); higher values are too high or toxic (according to Fürst (2018))

**Table 3:** Classification values for element ratios of *Alnus glutinosa*

S/N	N/P	N/Ca	N/Mg	N/K
0.035–0.080	8.3–26.7	1.7–13.3	8.3–26.7	1.7–4.0
K/Ca	K/Mg	Ca/Mg	P/Al	
0.7–5.0	3.3–10.0	1.0–10.0	12.5–29.1	

Ratios (dimensionless values): numbers in a given range are well balanced; values < lower limit are too low, values > upper limit are higher than the balanced value

Note: Results are determined from classification values (Table 2) as a ratio of the lower limit of the first element to the higher limit of the second one, a vice versa (Mellert & Göttlein, 2012)

### Statistical analysis

The obtained data were processed using STATISTICA (TIBCO Software Inc., 2018) and UNISTAT (Unistat Ltd., 2013) software. Data from the end of the growing season (element contents, selected ratios) were assessed using one-factor ANOVA (*H* = f(Loc)) at the factor levels 5 with the mean ± confidence interval and a Tukey HSD test, *α* = 0.05. A multidimensional analysis was also used, as well as the following cluster analysis methods: tree clustering, cluster row, complete linkage, and distance measure 1-Pearson r.

## Results

### The content of elements in soil

Data from direct field measurements were largely varied. Element contents in the soils and the differences between the Ah and B horizons were evaluated according to laboratory data. A *t*-test for dependent samples (variables Ah and B) was conducted with *n* = 15 (each variable) and *α* = 0.05 (TIBCO Software Inc., 2018). The contents of S, P, Ca, and Pb were significantly higher in the Ah horizon than in the B horizon, while the opposite was found for K and Al. Significant differences were not found for Mn, Zn, and Cu. For Mn, Zn, Cu, and Pb, classification according to the Soil Protection and Remedial Actions Criteria (Moen et al., 1986) was also used (Suppl. Table S2).

### The content of elements in leaves

#### Macroelements

The N content in *A. glutinosa* leaves varied during the growing season at the dry sites (HA, HH), decreasing trend prevailed at waterlogged sites (PE, SV, UC), (Fig. 1 N). During the late-growing season, the average N content in leaves at the humid sites (SV, UC) was > 30 mg g^−1^. Significant differences were observed between the dry site (HA) and the sites with moisture conditions more favorable for *A. glutinosa* growth (SV and UC) (Table 4). In terms of nutrition, the N content was adequate to optimal (Table 2).

**Table 4:** Content of elements in the leaves of *Alnus glutinosa* at the end of the growing season

Element^1a^	Loc^2^	Mean ± CI^3^	HSD^4^			Element^1b^	Loc^2^	Mean ± CI^3^	HSD^4^			
N	HA	26.0 ± 2.7	a			Mn	HA	184 ± 84	a			
	HH	27.0 ± 3.1	a	b			HH	390 ± 96	a			
	PE	28.0 ± 2.0	a	b			PE	769 ± 342		b		
	SV	31.2 ± 1.6		b			SV	450 ± 39	a	b		
	UC	31.3 ± 4.8		b			UC	445 ± 268	a	b		
S	HA	1.34 ± 0.04	a	b		Zn	HA	79 ± 52	a			
	HH	1.23 ± 0.24	a				HH	122 ± 39	a	b		
	PE	1.41 ± 0.10	a	b			PE	63 ± 16		b		
	SV	1.48 ± 0.06		b			SV	104 ± 14	a	b		
	UC	1.51 ± 0.08		b			UC	99 ± 6	a	b		
P	HA	1.09 ± 0.16	a			Cu	HA	9.41 ± 1.89	a			
	HH	1.17 ± 0.15	a				HH	11.48 ± 0.45	a	b		
	PE	1.36 ± 0.04	a	b			PE	14.20 ± 0.74		b		
	SV	1.45 ± 0.28		b			SV	11.34 ± 2.81	a			
	UC	1.44 ± 0.29		b			UC	10.84 ± 1.77	a			
Ca	HA	16.27 ± 1.52	a			Pb	HA	0.65 ± 0.32	a			
	HH	16.60 ± 3.97	a				HH	0.72 ± 0.05	a			
	PE	11.65 ± 1.47		b			PE	0.71 ± 0.21	a			
	SV	7.37 ± 1.08			c		SV	0.64 ± 0.03	a			
	UC	9.22 ± 2.63		b	c		UC	1.02 ± 0.45	a			
Mg	HA	2.56 ± 0.84	a	b		Cd	HA	0.056 ± 0.040		b		
	HH	1.90 ± 0.19	a				HH	0.099 ± 0.032	a			
	PE	2.40 ± 0.26	a	b			PE	0.052 ± 0.027		b		
	SV	2.48 ± 0.55	a	b			SV	0.021 ± 0.004		b		
	UC	3.02 ± 0.49		b			UC	0.029 ± 0.010		b		
K	HA	6.88 ± 1.45	a			Al	HA	252 ± 51	a			
	HH	6.70 ± 2.05	a				HH	190 ± 15		b		
	PE	6.10 ± 1.64	a				PE	64 ± 11			c	
	SV	5.64 ± 1.76	a				SV	135 ± 13				d
	UC	5.49 ± 0.88	a				UC	135 ± 49				d

^1a^Value [mg g^−1^]; ^1b^[value μg g^−1^]; ^2^see Table 1; ^3^CI confidence interval *α* = 0.05, *n* = 6; ^4^Tukey test significant difference; *α* = 0.05; different letters indicate a significant difference (data processing (Unistat Ltd, 2013))

The P content (Fig. 1 P) was highest at all sites at the beginning of the growing season and then decreased. At lower altitudes, P content stabilized during the early-growing season; at altitudes > 750 m a.s.l. (SV and UC), it declined from 3.3 to 3.9 mg g^−1^ until the end of the early-growing season when the level of 2 mg g^−1^ was reached. The lowest values were determined at the end of the late-growing season. Significant differences were observed between HA, HH × SV, and UC sites (Table 4). The P contents at sites HA, HH, and PE were low/deficient, while those at the other sites were at the upper limit (Table 2). In general, the P content in the stands was low.

The S content of the *A. glutinosa* leaves was stable during the growing season. Higher content of S found during the early-growing season at altitudes > 600 m decreased to the level comparable with other stands before the fast-growing season (Fig. 1 S). During the late-growing season, average S content in the leaves decreased slightly, except PE, where it increased to a level comparable with other stands. The differences between HH × SV and UC were significant (Table 4). The values are in the range of adequate to optimum content levels (Table 2).

From the start of the early-growing season, the Ca content in *A. glutinosa* leaves was increasing at all sites and the differences were significant. In wet and waterlogged sites (PE, SV, UC), where the soil Ca content was generally lower (1249–5,998 mg kg^−1^; Suplementary material Table S2), the increase in leaves was varied, but did not exceed 12 mg g^−1^. Higher levels were found at dry sites (HA, HH; soil Ca content 3412–8183 mg kg^−1^; Suplementary material Table S2) during the fast-growing season (Fig. 1 Ca). In the late-growing season, average Ca content in leaves was increased in dry sites HA and HH, but sufficient to optimal at other sites in terms of nutrition. Significant differences were determined between the sites HA, HH × PE, SV, UC, and PE × SV (Tables 2 and 4).

The Mg content in the *A. glutinosa* leaves was varied, and max. value was obtained at the UC site (2.7–3.4 mg g^−1^) (Fig. 1 Mg); the results at the end of the growing season varied as follows: (HH < HA, PE, SV < UC). Mg content level was elevated at the UC site and sufficient in all other stands. Mg concentrations at the sites HH and UC were significantly different (Tables 2 and 4).

The K content in *A. glutinosa* leaves decreased during the growing season (Fig. 1 K) to low levels (< 6 mg g^−1^ for stands on waterlogged soils). In terms of nutrition, the content of K was insufficient. Differences between the sites were insignificant (Tables 2 and 4).

#### Microelements

**Fig. 2:**
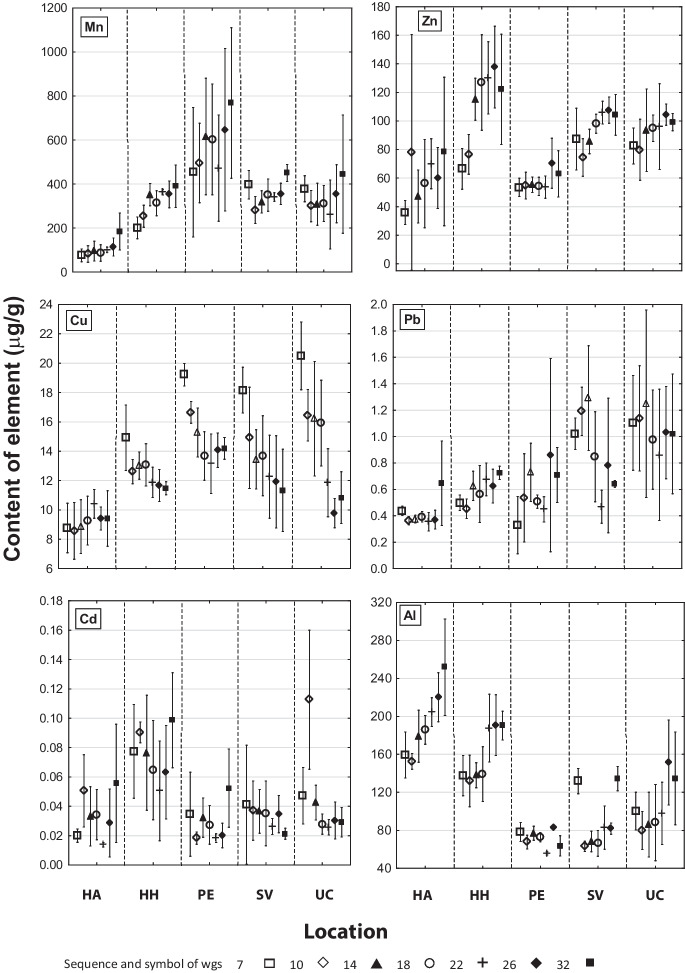
Content of microelements and non-nutrient elements in *Alnus glutinosa* (mean ± confidence interval; according to the week of growing season - “wgs”)

The content of Mn in *A. glutinosa* leaves increased at sites HH, HA, and PE or varied (SV and UC) during the growing season (Fig. 2 Mn). During the late-growing season, Mn content was higher in wet soils (> 400 μg g^−1^) with max. value 760 μg g^−1^ at the waterlogged site PE, despite the lower total Mn content in the soil (52–142 mg kg^−1^). Low levels (< 200 μg g^−1^) were observed in leaves at the dry site HA (Fig. 2 Mn), where Mn content in soil was the highest (417–462 mg kg^−1^, Suplementary material Table S2). In terms of nutrition, there was surplus Mn content, and significant differences were found between HA and HH × PE (Tables 2 and 4).

The content of Zn in the *A. glutinosa* leaves (Fig. 2 Zn) varied during the growing season, but in general, it increased; at the dry soil of site HH (increased soil Zn content 186 mg kg^−1^), the increase was more significant in the early-growing season than in the other ones. During the late-growing season, the highest content was found in leaves from HH (> 120 μg g^−1^); the other stands showed < 100 μg g^−1^ at soil contents of 43–67 mg kg^−1^ (Table S2). A significant difference was determined between HA × PE values (Table 4). The Zn content was excessive from the nutritional point of view (Fig. 2 Zn; Tables 2 and 4, Suplementary material Table S2).

The content of Cu in the *A. glutinosa* leaves was constant at the HA site, but showed a decreasing trend at higher elevations during the late-growing season (Fig. 2 Cu). At the end of the growing season, the concentration was ≤ 12 μg g^−1^ except PE with > 14 μg g^−1^ (higher Cu content in soil horizon A, Suplementary material Table S2). Significant differences were found between PE × HA, SV, and UC values. As for nutrition, Cu content was sufficient to excessive (Fig. 2; Tables 2 and 4).

#### Non-nutrient elements

The content of Pb in *A. glutinosa* leaves mostly varied during the growing season, higher levels were determined at the wet sites (SV, UC; Fig. 2 Pb), up to < 1 μg g^−1^ or almost 1 μg g^−1^ (UC) during the late-growing season. The differences were not significant. In terms of nutrition, Pb content was low, adequate to the background (Fig. 2 Pb; Tables 2 and 4).

The content of Cd in *A. glutinosa* leaves varied at all sites; during the late-growing season, it increased at HA, HH, and PE and decreased at SV and UC sites (Fig. 2 Cd). At the end of the growing season, the values varied in the range of 0.02–0.06 μg g^−1^ except for HH (almost 0.1 μg g^−1^, significantly different from all the other sites). Similarly, to Pb, the Cd content was low in terms of nutrition, adequate to the background level (Fig. 2 Cd; Tables 2 and 4).

Higher content of Al, further increasing during the growing season, was observed in the *A. glutinosa* leaves at the dry sites HA and HH, while lower, varied content was measured at the sites affected by water (PE, SV, and UC) (Fig. 2 Al). At the end of the growing season, low concentration was found at the wetland site PE (ca. 60 μg g^−1^), high moisture sites SV and UC showed > 120 μg g^−1^, and a significantly higher, toxic level was determined at HH (190 μg g^−1^) and HA (252 μg g^−1^) sites. Leaf damage symptoms were not observed. Significant differences were found between HA, HH, PE × SV, and UC sites (Fig. 2 Al, Tables 2 and 4).

### Mutual ratios of most important elements

**Fig. 3:**
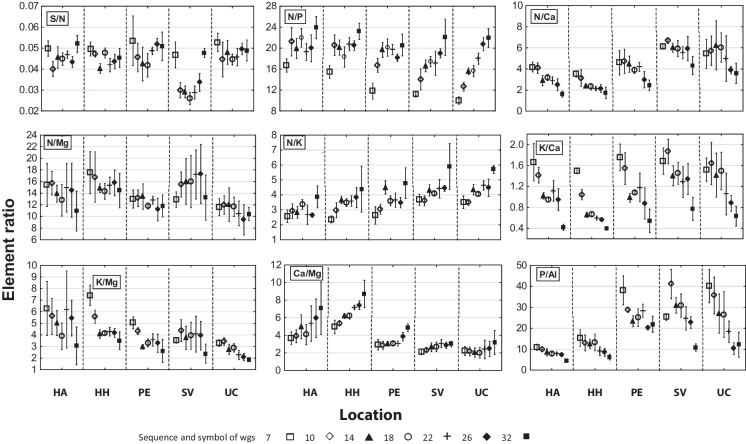
Selected ratios of elements in the leaves of *Alnus glutinosa* (mean ± confidence interval; according to the week of growing season - “wgs”

The S/N ratio varied during the growing season (Fig. 3 S/N), but showed comparable values in the early- and late-growing season. From the viewpoint of S and N content, the ratio was balanced at the end of the growing season, with no significant differences among the sites (Tables 3 and 5).

**Table 5:** Ratios of selected element in leaves of *Alnus glutinosa* at the end of the growing season

Ratio	Loc^1^	Mean ± CI^2^	HSD^3^		
S/N	HA	0.052 ± 0.004			
	HH	0.045 ± 0.004			
	PE	0.051 ± 0.007			
	SV	0.048 ± 0.002			
	UC	0.050 ± 0.005			
N/P	HA	23.92 ± 2.12			
	HH	23.22 ± 1.58			
	PE	20.58 ± 2.09			
	SV	22.06 ± 3.48			
	UC	21.96 ± 1.79			
N/Ca	HA	1.61 ± 0.28	a		
	HH	1.72 ± 0.53	a		
	PE	2.45 ± 0.49	a		
	SV	4.32 ± 0.85		b	
	UC	3.58 ± 0.98		b	
N/Mg	HA	10.92 ± 3.41			
	HH	14.45 ± 2.94			
	PE	11.80 ± 1.89			
	SV	13.24 ± 3.87			
	UC	10.71 ± 1.11			
N/K	HA	3.87 ± 0.42	a		
	HH	4.38 ± 1.53	a	b	
	PE	4.79 ± 1.02	a	b	
	SV	5.89 ± 1.53		b	
	UC	5.70 ± 0.25		b	
K/Ca	HA	0.42 ± 0.06	a		
	HH	0.40 ± 0.03	a		
	PE	0.54 ± 0.22	a	b	
	SV	0.77 ± 0.22		b	
	UC	0.63 ± 0.19	a	b	
K/Mg	HA	3.05 ± 1.62	a	b	
	HH	3.49 ± 0.77	a		
	PE	2.61 ± 0.99	a	b	
	SV	2.37 ± 0.82	a	b	
	UC	1.82 ± 0.13		b	
Ca/Mg	HA	7.09 ± 2.99	a		
	HH	8.71 ± 1.55	a		
	PE	4.86 ± 0.45	a	b	
	SV	3.02 ± 0.31		b	
	UC	3.18 ± 1.35		b	
P/Al	HA	4.41 ± 0.70	a		
	HH	6.20 ± 1.33	a	b	
	PE	21.89 ± 3.82			
	SV	10.75 ± 1.74		b	c
	UC	12.12 ± 5.96			c

^1^See Table 1; ^2^CI confidence interval *α* = 0.05, *n* = 6; ^3^Tukey test significant difference; *α* = 0.05; different letters indicate a significant difference (data processing (Unistat Ltd, 2013))

At the sites with higher moisture (PE, SV, and UC; FSC G), a low N/P ratio (< 14) during the early-growing season indicated lower N uptake compared to P. Later, the ratio increased (> 16), especially at the wet sites SV and UC (Fig. 3 N/P), and the trees had sufficient N, but limited P uptake. No significant differences were determined among the sites (Tables 3 and 5).

The N/Ca ratio continually declined during the growing season due to the decreasing content of N and increasing content of Ca in leaves (Fig. 3 N/Ca). At the drier sites, HA and HH, the ratio reached the lower limit of balanced value, while at other sites the values belonged to the lowest 25% of the balanced value interval. Data found for HH, HA, and PE sites were significantly different from SV and UC locations (Tables 3 and 5).

The N/Mg ratio varied with no trend during the growing season (Fig. 3 N/Mg). Tree nutrition was balanced, with no significant differences among the sites (Tables 3 and 5).

The N/K ratio was continually increasing during the growing season (Fig. 3 N/K). Except for a balanced value at HA, ratios close to the upper limit for balanced values were found at all sites. The results from the HA site were significantly different from SV and UC (Tables 3 and 5).

The K/Ca ratio showed a decreasing trend (Fig. 3 K/Ca) with the lowest value at the end of the growing season. The ratio was lower than balanced at HA and HH and almost balanced at high moisture sites. In general, the ratio was not balanced due to insufficient uptake of K. HA and SV sites differed significantly from each other (Tables 3 and 5).

The K/Mg ratio was varied with no obvious trend during the growing season, although in the late-growing season, the values were lower than in the early-growing season (Fig. 3 K/Mg). Except for HH (balanced), ratios at the other sites were in the lower balanced category due to low K uptake. Values from HH and UC sites differed significantly from each other (Tables 3 and 5).

The Ca/Mg ratio increased significantly during the growing season at the dry sites HA and HH and was balanced in the high moisture stands; the highest values were obtained at the end of the growing season (Fig. 3 Ca/Mg). In terms of nutrition, the ratios were more balanced at HA and HH and less at PE, SV, and UC sites. Significant differences were found between HA, HH × SV, and UC results (Tables 3 and 5).

The P/Al ratios varied (PE and SV), mostly decreased (HA, HH, and UC), and were lower at the end of the growing season compared to the beginning (Fig. 3 P/Al). The changes were influenced by the Al content in the leaves. At the end of the growing season, the ratio was low for HA and HH and medium level for SV and UC sites. Permanently waterlogged PE site showed a high value due to low Al content (Table 5). In terms of nutrition, balanced values were obtained from PE and lower balanced ratios from all other sites (Table 3).

### Cluster analysis of the element contents

**Fig. 4:**
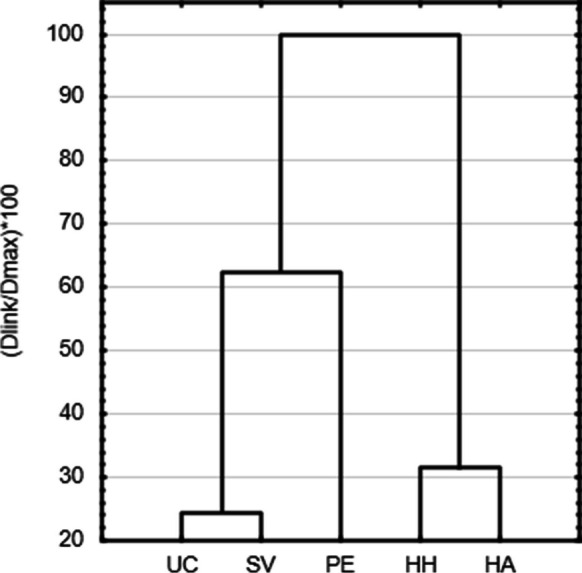
Similarity of leaf element content levels at individual sites (cluster analysis)

Cluster analysis was used to evaluate the element content levels in the leaves at all sites, using the data from the end of the growing season. The objects created represent individual sites characterized by all 12 variables (elements observed in leaves). The clusters were consistent with the forest site complex. The first group consisted of the most similar sites SV and UC, joined by a less similar PE (all FSC G). The second group with close similarity consisted of HA and HH sites (Fig. 4).

## Discussion

### The content of individual elements in soil

The risks of determining the soil element content by XRF measurements “in situ” were reported by Menšík et al. (2021); the accuracy is affected by moisture, porosity, and soil texture of the field samples (Padilla et al., 2019; Sahraoui & Hachicha, 2017). To prevent such influence, data from laboratory measurements were used for the evaluation.

Macroelements and Al are natural components of soil; classification of their content is pointless. The unbalanced content of the macroelements could be determined by visual assessment of the health condition of the stands. The contents of Mn, Zn, and Cu microelements corresponded to their normal occurrence in soils and only the Pb content was above average at all sites (Kabata-Pendias, 2011). For a more detailed assessment, a classification according to the Soil Protection and Remedial Actions Criteria (Moen et al., 1986) was conducted. This classification confirmed the increased content of Pb and Cu (only in Ah at the PE site).

Differences in the relative content of elements in studied horizons can be explained by the hypothesis that compared to other horizons. The organic-mineral Ah horizon with higher organic matter content and pH retained more S, P, Ca, and Pb compared to horizon B. Higher K and Al deposits were in horizon B, as they were released more easily from primary minerals.

### The content of individual elements in leaves

Nutrient dynamics is element-specific during the growing season. In the beginning, when leaves are forming and metabolic processes are initiated, nutrient consumption is high. In the early-growing season (May–June), the leaves are fully developed and the content of elements in the leaves decreases. During the fast-growing season (July–September), the amount of elements in the leaves is constant or slightly varied while the tree is in a stage of stable growth and both uptake and consumption of nutrients are balanced. During the late-growing season (October), the amount of elements in leaves depends on transport related to leaf aging mechanisms (Lambers et al., 2008).

#### Macroelements

Biological fixation from symbiosis with the bacteria *Frankia* sp. is almost the only source of N uptake for *A. glutinosa* (Roy et al., 2007). During the growing period, the transport and accumulation of N to other parts of a tree is based on the high mobility of nitrogenous compounds (Marschner, 2006). Despite variation among the values, a general trend of continual N content decrease was confirmed from the beginning to the end of the growing season; these results are in good accordance with the findings of Yan et al. (2016) about deciduous trees. The apparent increase in the N content during the early-growing season at individual sites (a higher effect was observed with increasing altitude) can be explained by the gradual development of foliage and its function. According to our opinion, fully developed leaves (visual evaluation) do not guarantee full possible capacity performance.

The content of N in *A. glutinosa* was adequate at the end of the growing season; according to Göttlein and Zehle (2018), the values were in a normal range. Our hypothesis suggests that differences between N content values determined in leaves during and at the end of the growing season correspond to the average intensity of N fixation by actinorhizae (comp. Millett et al. (2012)) and low supply of N compounds from emissions in the monitored area. The contents of N determined in *A. glutinosa* leaves were comparable to or higher than the ones in other deciduous trees (Göttlein & Zehle, 2018; Yan et al., 2016).

Roots in the form of sulfate assimilate S, leaves can uptake small amounts of SO_2_. Adequate (or excessive) input S compounds during the growing season are stored as sulfate, which is transported from older to younger leaves (Marschner, 2006). Changes in S content in *A. glutinosa* leaves during the growing season were related to their actual root uptake as indicated by the low content of S at the SV site; however, this mechanism did not explain the increased uptake of S compounds in the middle of the growing season (at low immission conditions). The level of S in the leaves was reported to reflect the environmental burden from past immissions still present in the stands, as shown for example by the data for *B. pendula* (Hrdlička & Kula, 2009). From the nutrition viewpoint, the sulfur content level in *A. glutinosa* ascertained at the end of the growing season was adequate.

The uptake of P compounds was higher at the beginning of the growing season. The initial amount of P in the leaf is high, especially in newly developed leaves, ensuring the start of the growing season; then, the P content decreases in fully (morphologically and functionally) developed leaves due to reduced P uptake and increased leaf weight. During the late-growing season, a decrease was reported during senescence (Lambers et al., 2008; Li et al., 2017; Yan et al., 2016). The obtained results are in good accordance with that, the content of P in *A. glutinosa* leaves was low at the end of the growing season. In general, the content of P in leaves was low and deficient compared to both alder (Göttlein & Zehle, 2018) and other deciduous tree species (Göttlein & Zehle, 2018; Mellert & Göttlein, 2012). The results were affected also by stand location within the FSC; drier HA and HH sites had lower P supply than PE, SV, and UC locations with moderately rich, more moisture soils.

Ca is typically an immobile element (Marschner, 2006) that cannot be transported from older to young leaves (White & Broadley, 2003). It is stored in leaves and the content was reported to increase during the growing season (Lambers et al., 2008; Yan et al., 2016), as confirmed by our results. The content of Ca in *A. glutinosa* determined at the end of the growing season was adequate, the values were within the normal range according to Göttlein and Zehle (2018). A clear difference was observed in the stand nutrition between HA and HH sites (higher Ca content) and all other sites. The needs of *A. glutinosa* are greater than those of other deciduous tree species (Göttlein & Zehle, 2018; Mellert & Göttlein, 2012).

Both K and Mg are high-mobility elements (Marschner, 2006). The amount of Mg in leaves showed a general variability trend during the growing season, but not the trend of increasing Mg content reported by Yan et al. (2016). The amount of K in leaves showed a general decreasing trend with leaves containing the lowest amounts of K at the late-growing season (in good accordance with Yan et al., 2016) except for the dry site HA where the values varied during the growing season. In terms of nutrition, the Mg content was adequate in *A. glutinosa* leaves, but K was deficient. Similar results were reported for *A. glutinosa* and other deciduous tree species (Göttlein & Zehle, 2018; Mellert & Göttlein, 2012; Yan et al., 2016).

#### Microelements

Mobility of Mn is limited and its amount in the leaves of woody plants increases during the growing season (Marschner, 2006). Our results support these findings; despite the fact that the values were slightly varied during the growing season, Mn content was the highest in *A. glutinosa* at the end of the growing season. Different values in monitored areas are related to their soil moisture (e.g., Wildová et al., 2021), and generally correspond to forest site complex. From the viewpoint of stand nutrition, Mn content was high at all sites, > 100 μg g^−1^. According to Göttlein and Zehle (2018), the values are in the normal range. These results further support our hypothesis that limit values for Mn in deciduous tree leaves were underestimated, as shown by other sources (e.g., Göttlein & Zehle, 2018). Some forest tree species and plants on the forest floor tend to hyperaccumulate Mn without showing signs of damage. For *B. pendula*, for example, leaf content values varied in the range 118–9743 μg g^−1^ (Hrdlička & Kula, 2004), 284–1790 μg g^−1^ (Wisłocka et al., 2006), or 6956–10,852 μg g^−1^ (Wildová et al., 2021); for *Larix decidua* Mill., the range was 9689–13,508 μg g^−1^, and for *Vaccinium myrtillus* L., 5982–12,172 μg g^−1^ (Wildová et al., 2021). These values exceed the established toxicity limit of 400–1000 μg g^−1^ (Kabata-Pendias, 2011), but visually the *A. glutinosa* stands studied did not show any signs of poisoning from excess Mn toxicity.

Mobility of Zn is moderate and transport from old to young leaves was observed (Marschner, 2006). Our results are in good accordance with those findings; increasing Zn content was observed during the growing season. However, Yan et al. (2016) reported lower values of Zn content and a decrease in deciduous tree leaves. In this study, the content of Zn found in the leaves at the end of the growing season was high, but according to Göttlein and Zehle (2018) still in the normal range. According to the literature, Zn content in other deciduous trees is in the normal range (Göttlein & Zehle, 2018), or excessive (Yan et al., 2016), but not above the phytotoxicity limit of 250 μg g^−1^ (Linzon et al., 1976).

Cu has moderate mobility similar to that of Zn (Marschner, 2006). Above the critical level, which is 40 μg g^−1^ (Linzon et al., 1976) or 100 μg g^−1^ (Cicek & Koparal, 2004) in leaves, Cu is a toxic element. In monitored stands, the content of Cu in *A. glutinosa* leaves decreased gradually during the growing season. Unlike other less mobile elements, Cu is not accumulated in the leaves of forest tree species (Dmuchowski & Bytnerowitz, 1995). It is stored in roots and transported into the leaves only when necessary (West, 1979). From the viewpoint of nutrition, the Cu content in leaves was adequate; according to Göttlein and Zehle (2018), the values were in the normal range. The stands were well supplied with Cu compounds, but safely below the reported toxicity threshold.

#### Non-nutrient elements

Pb is found naturally in plants but it does not play an essential role in their metabolism. Generally, Pb is a toxic element (Kabata-Pendias, 2011), with critical levels in leaves reported to be 20 μg g^−1^ and lethal levels 40 μg g^−1^ (Burton & Morgan, 1983), or even 30 μg g^−1^ (Cicek & Koparal, 2004). During the growing season, Pb content in *A. glutinosa* leaves was varied, increasing at HA and HH sites during the last growing season and decreasing slightly in all other sites. These results are interesting compared to Godzik (1993), who reported the highest Pb content in leaves before the last growing season. At the end of the growing season, the Pb content in leaves was < 1 μg g^−1^, which is the background value (the sites were not contaminated with Pb compounds). Pb content in *A. glutinosa* leaves determined in this study at the end of the growing season is comparable to birch but higher than concentrations reported for aspen and maple (McGee et al., 2006, 2007), and a typical Pb content in leaves of deciduous trees is up to 0.8 μg g^−1^.

Cd is a toxic element (Marschner, 2006), and its critical level in leaves is reported to be 5 μg g^−1^ (Cicek & Koparal, 2004; Linzon et al., 1976). The estimated mobility of Cd is moderate, lower than that of otherwise similar Zn due to easy binding to -SH groups. Cd deposition in leaves depends on the actual situation in stands; more Cd was found in leaves at HA, HH, and PE sites than in SV and UC. The Cd content in *A. glutinosa* leaves measured in this study at the end of the growing season was < 1 μg g^−1^, which corresponded to background values (studied sites are not contaminated). At the same time, Cd values were significantly lower than those reported for aspen, birch, and maple (McGee et al., 2006, 2007).

Plants can contain up to 200 μg g^−1^ Al (Mossor-Pietraszewska, 2001). The estimated mobility of Al is low and, as our results suggest, depends on the potential for its uptake. At the acidic, dry HA and HH sites, the Al content in leaves was higher and was further accumulated during the growing season; at waterlogged (PE) and wet (SC, UC) sites, the amount of Al in the leaves was low and the values varied during the growing season. At the end of the season, the Al content in *A. glutinosa* leaves was high in all sites except PE, where the value was equal to the background level (60 μg g^−1^) due to its limited uptake potential in a permanently waterlogged habitat. At the acidic sites HA and HH, Al content exceeded the normal value and could be considered as possibly toxic (Mossor-Pietraszewska, 2001). However, symptoms of stand damage were not observed.

### Mutual ratio of selected elements

The S/N ratio varied during the growing season due to the actual uptake of these elements by vegetation according to their availability in soil stock and emission input. Atmospheric inputs were very low in the summer months; the S/N ratio at the SV site showed a low supply of sulfur compounds from the soil in the middle of the growing season and reduced content of S in the leaves. Since N is a priority for growth (Fageria, 2001), it is clear that low values of the S/N ratio are caused by low S supply. At the end of the growing season, the S/N ratio stabilized in the range of 0.045–0.052 (balanced values according to the classification, but lower than the reported optimum value of 0.077 (Baronius et al., 1997)).

The N/P ratio increased even in cases when the N content in the leaves actually decreased, because the P content decreased more significantly. At the beginning of the growing season, when young leaves were developing, the ratio decreased, because the P requirement was higher than the corresponding N uptake. At PE, SV, and UC sites (FSC G), the results were < 12, which is a value reported for young leaves with growth limited by nitrogen uptake (Koerselman & Meuleman, 1996). Later in the growing season, the uptake of P compounds increased according to the tree needs, to the level balanced with N uptake. The increase of the N/P ratio in leaves was caused also by the fact that P compounds are transported mostly to permanent parts of the tree, while N uptake is performed directly by the roots, thanks to the actinorhizal nature of *A. glutinosa* (Côte et al., 1989). Thus, in general, the N/P ratio increases as a result of a decrease in the amount of P in leaves. At the end of the growing season, the N/P ratio was balanced with values > 20, i.e., in the upper third of the range. P compounds were transferred from the leaves, which was related to senescence (Güsewell, 2004).

The N/Ca ratio decreased gradually during the growing season, in line with decreasing N content and increasing Ca content in leaves. At the end of the growing season, the results from HA and HH sites were equivalent to the lowest balanced values, but PE, SV, and UC sites were clearly above. These results were consistent with FSC characterization of the sites (Viewegh et al., 2003) and higher N content in leaves at HA and HH sites. The values found were higher compared to those reported for seedlings (Kuznetsova et al., 2010; Lorenc-Plucińska et al., 2013) due to the lower N uptake capacity of young plants. Values obtained for *A. glutinosa* were similar to those reported for other deciduous trees (Mellert & Göttlein, 2012; Stefan et al., 1997).

The N/K ratio increased during the growing season due to a relatively larger decrease in K content in the leaves. At the end of the growing season, the results were above the upper limit for balanced values. Similar findings have been described for beech (Mellert & Göttlein, 2012).

The decreasing trend of the K/Ca ratio corresponded to decreasing K content and increasing Ca content. At the end of the growing season, all sites showed adequate or lower balanced values. Increased Ca content with decreasing K content was more apparent at HA and HH sites—values of Ca were higher and K too low. Similarly, the trend of decreasing K/Mg values corresponded to a decreasing K content, despite varied amounts of Mg in leaves. At the end of the growing season, the results were ≤ the limit for balanced values, indicating K deficiency, which was higher at PE, SV, and UC (FSC G) sites.

The increase in the Ca/Mg ratio was caused by increasing Ca content; Mg content was varied. At the end of the growing season, the results were balanced, although different at HA, HH sites (FSC H and N) and SV, UC sites (FSC G). All locations had a sufficient supply of both elements in accordance with the needs of the trees.

As for the P/Al ratio, the content of P was generally decreasing and the changes were caused by varied Al content in the leaves. At the end of the growing season, the ratios at sites PE (higher value) and UC (lower value) were balanced (normal content values), while results from HA and HH (significantly low values) and SV (higher value) sites were in the lower balanced range. The results indicate a possible negative influence to P uptake due to the effect of Al uptake.

### Cluster analysis for the content of elements

The results of the cluster analysis for element content corresponded to the characteristics of the sites according to the FSC (Viewegh et al., 2003). The PE site (FSC: 6G) showed different results due to permanent waterlogging, SV and UC (FSC: 7G and 8G) sites were characterized by a high water level, and HA (2H) and HH (4 N) sites were characterized by reduced moisture conditions (Fig. 4).

## Conclusion

The current state of knowledge on the development of element content levels in *A. glutinosa* leaves during the growing season is limited. Thanks to the specific N fixation mechanism, this tree species has unique characteristics compared to other tree species in the family Betulaceae (*Betula*, *Carpinus*, and *Corylus*). *Alnus glutinosa* stands were studied at five sites in the Ore Mountains (north Czech Republic) with different altitudes and habitat conditions, including differentiated soil and precipitation or moisture conditions.

The total content of elements in the soil of the sites corresponded to the natural background except the content of Pb in Ah horizon at the wet sites (PE, SV, UC), which was classified as medium contamination. A difference was found for each element between the Ah and B horizons, which is consistent with their nature.

The contents of macronutrients (N, S, P, Ca, Mg, and K), micronutrients (Mn, Zn, and Cu), and non-nutrient elements (Pb, Cd, and Al) in the leaves were monitored during one growing season (May to October 2009). Considering the 550 m altitude difference between HA and UC sites, there were apparent differences in foliage phenology and the leaves’ development during the spring, which partly influenced variations in the development of some element content (N, P, and Cu). Seasonal dynamics of each element’s content corresponded to their importance for the growth of *A. glutinosa*. Despite variations, a general trend of decreasing content was found for N, S, P, K, Cu, and Cd. The contents of Ca, Mn, Zn, and Al generally increased, while the contents of Mg and Pb varied with no trend.

Fulfillment of nutritional requirements of *A. glutinosa* was assessed according to the elemental content at the end of the growing season. The N content was adequate, corresponding to the average intensity of N fixation due to actinorhizae and the minimal supply of N compounds from emissions. Appropriate content level was observed also for S, Ca, Mg, and Cu, while for P and K it was low and for Mn and Zn too high. The content of non-nutrient elements Pb and Cd corresponded to background values; the content of Al was similar to the background level (waterlogged site PE), medium (sites SV and UC), or high (dry sites HA, HH). Element to N ratios were mostly balanced and only S/N was decreasing (low S and high N content). Due to K deficiency, N/K ratio was high, while K/Ca and K/Mg ratios were low. The Ca/Mg ratio was also balanced, P/Al ratio showed varied values. Cluster analysis results for the elements were consistent with those for the forest site complex; the clusters were related according to the amount of water in the soil.

### Supplementary information

ESM 1 (ZIP 242 kb)

## Data Availability

The datasets generated and analyzed during the current study are available from the corresponding author on reasonable request.
