# Supplementary material for: Element contents and their seasonal dynamics in leaves of alder Alnus glutinosa (L.) Gaertn
Source: Environ Monit Assess. 2024 Feb 1;196(2):224. doi: 10.1007/s10661-024-12367-x (PMC10834585; doi:10.1007/s10661-024-12367-x)
Supplement: Supplementary file 1 — (ZIP 242 kb) [file 10661_2024_12367_MOESM1_ESM.zip › EMS_1_Fig.pdf]

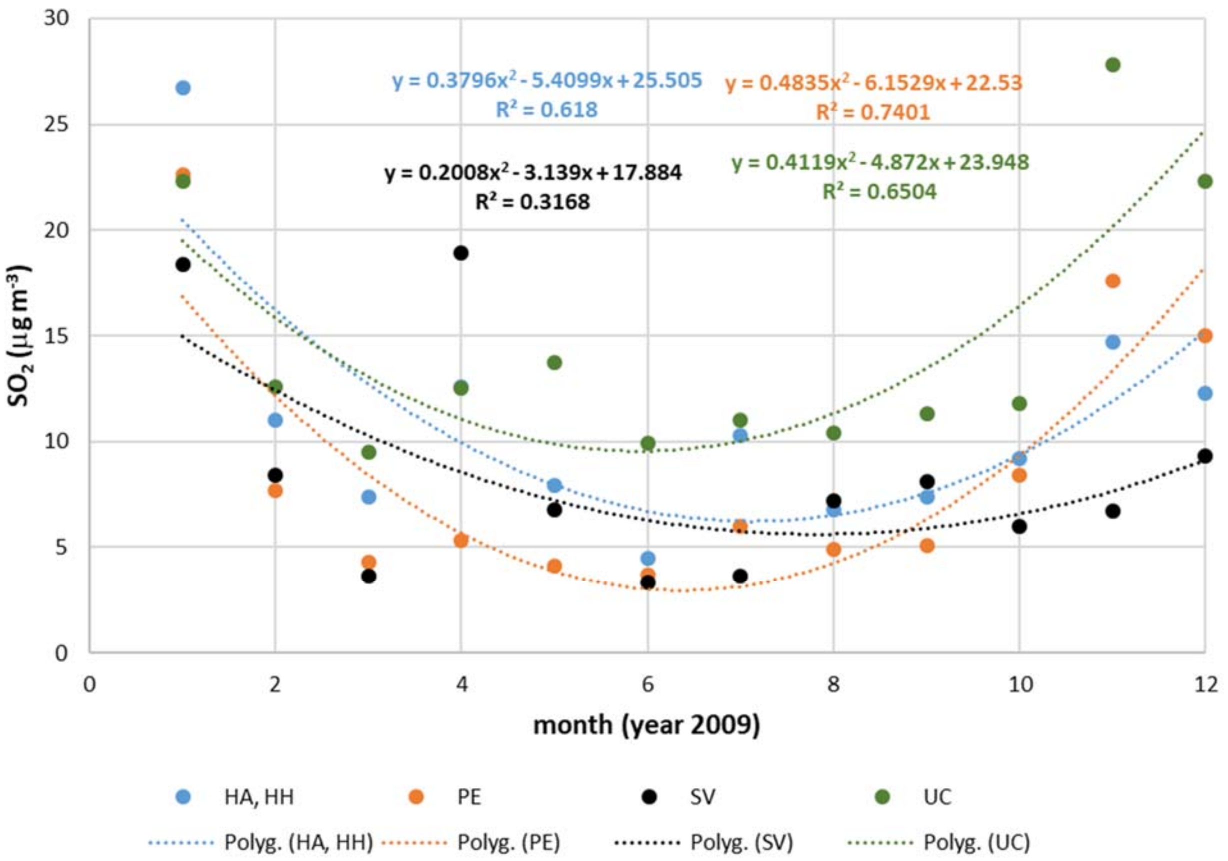

Fig 1a: Content of atmospheric SO_2 (μg m^{-3} $month^{-1}$) during the year 2009

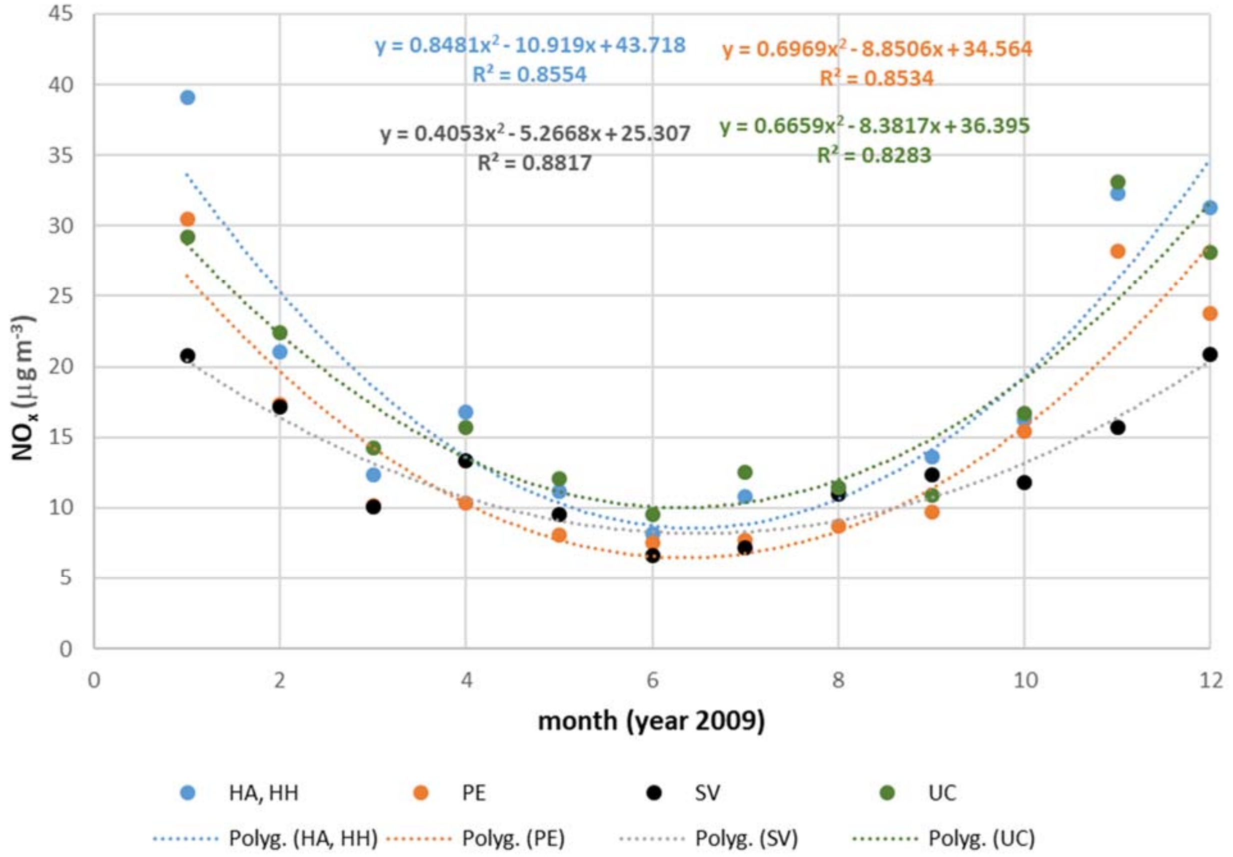

Fig 1b: Content of atmospheric NOx (μg m^{-3} $month^{-1}$) during the year 2009
