# Supplementary material for: Element contents and their seasonal dynamics in leaves of alder Alnus glutinosa (L.) Gaertn
Source: Environ Monit Assess. 2024 Feb 1;196(2):224. doi: 10.1007/s10661-024-12367-x (PMC10834585; doi:10.1007/s10661-024-12367-x)
Supplement: Supplementary file 1 — (ZIP 242 kb) [file 10661_2024_12367_MOESM1_ESM.zip › EMS_2_table_S1.pdf]

Table S1: Annual mean atmospheric $SO_2$ and $NO_x$ ($\mu g\ m^{-3}\ year^{-1}$) concentrations in localities of study (Czech Hydrometeorological Institute, 2011). Five-year trend.

| Loc | HA, HH | | PE | | SV | | UC | |
|---|---|---|---|---|---|---|---|---|
| Year | $SO_2$ | $NO_x$ | $SO_2$ | $NO_x$ | $SO_2$ | $NO_x$ | $SO_2$ | $NO_x$ |
| 2005 | 10.9 | 20.0 | 11.0 | 18.6 | 10.7 | 15.1 | 16.0 | 12.3 |
| 2006 | 15.2 | 22.4 | 13.0 | 17.4 | 9.5 | 15.1 | 18.4 | 13.5 |
| 2007 | 14.0 | 17.8 | 10.2 | 15.1 | 7.7 | 14.3 | 15.8 | 18.6 |
| 2008 | 10.1 | 18.8 | 9.3 | 16.0 | 7.4 | 12.4 | 14.2 | 19.3 |
| 2009 | 10.9 | 18.7 | 8.8 | 14.8 | 8.3 | 13.0 | 14.6 | 18.1 |

Marks from Table 1. Data adopted from stations: HA, HH - Lom 265 m a.s.l.), PE - Sneznik (588 m a.s.l.), SV - Rudolice v Horach (843 m a.s.l.), UC – Komari Vizka (774 a.s.l.)

*Czech Hydrometeorological Institute: Air Quality Protection Division* (2011). Annual Tabular Overview. http://www.chmi.cz/files/portal/docs/uoco/isko/tab_roc/tab_roc_CZ.html. [accessed 20.4 2011].
