# Supplementary material for: Element contents and their seasonal dynamics in leaves of alder Alnus glutinosa (L.) Gaertn
Source: Environ Monit Assess. 2024 Feb 1;196(2):224. doi: 10.1007/s10661-024-12367-x (PMC10834585; doi:10.1007/s10661-024-12367-x)
Supplement: Supplementary file 1 — (ZIP 242 kb) [file 10661_2024_12367_MOESM1_ESM.zip › EMS_3_table_S2.pdf]

Table S2: Soil element content (mg kg^{-1})

site	S_Ah	S_B	P_Ah	P_B	Ca_Ah	Ca_B	K_Ah	K_B
HA	772	540	2714	2601	8025	8183	16877	17781
HH	2048	1203	2786	2731	4003	3412	13509	16444
PE	3563	0	3807	1730	5998	2458	7666	21259
SV	1747	382	2989	2291	3983	2229	11749	14610
UC	2065	637	3065	2595	2409	1249	11587	15218

site	Mn_Ah	Mn_B	Zn_Ah	Zn_B	Cu_Ah	Cu_B	Pb_Ah	Pb_B	Al_Ah	Al_B
HA	417	462	128	151	30	34	46	61	31600	36633
HH	243	254	186	186	42	49	97	110^{B}	26100	31000
PE	52	142	67	63	62^{B}	19	257^{B}	33	29133	46833
SV	107	115	67	43	39	0	164^{B}	63	29667	34267
UC	128	37	66	56	38	17	241^{B}	115^{B}	35967	47467

Horizon Ah, Horizon B, determined in laboratory by XRF method
Dutch level (Moen et al. 1986): A – uncontaminated or backround; B – medium contamination; C – high or toxic contamination. Marked only B, others are A. Valid for Mn, Zn, Cu, Pb

Moen, J. E. T., Cornet, J. P., & Evers, C. W. A. (1986). Soil Protection and Remedial Actions: Criteria for Decision Making and Standardization of Requirements. In J. W. Assink & W. J. Van Den Brink (Eds.), *Contaminated Soil: First International TNO Conference on Contaminated Soil 11–15 November, 1985, Utrecht, The Netherlands* (pp. 441-448). Springer Netherlands. https://doi.org/https://doi.org/10.1007/978-94-009-5181-5_54
